# Transient Hypophosphatemia: A Dangerous Event in Multiple Myeloma

**DOI:** 10.1155/2021/3609346

**Published:** 2021-01-16

**Authors:** Paula Jeffs, Michelle Mangual-García, Alex González-Bóssolo, Nadyeschka Rivera-Santana

**Affiliations:** Endocrinology Department, San Juan City Hospital, San Juan, PR 00921, USA

## Abstract

Patients with malignancies frequently experience multiple electrolyte disturbances. In multiple myeloma, hypercalcemia and hyperphosphatemia are one of the most common metabolic disturbances observed as part of pathogenesis of the disease. However, in rare occasions and during the course of the disease, many patients can develop hypophosphatemia due to multiple factors that affects the phosphate absorption and excretion. We hereby present a 56 y/o woman recently diagnosed with multiple myeloma who developed severe hypophosphatemia during medical treatment. We should note that the following manuscript was presented at the 2019 American Association of Clinical Endocrinologists (AACE) 28th Annual Scientific and Clinical Congress.

## 1. Introduction

Cancer is the second cause of death in the United States, and multiple myeloma accounts for approximately 1 to 2 percent of all cancers [1]. In such regard, the American Cancer Society estimates that, in 2020, new cases of multiple myeloma will reach 32,270 in the U.S., while deaths could top 12,830 [2]. This disease is characterized by neoplastic proliferation of immunoglobulin-producing plasma cells, and patients may present a variety of signs or symptoms including, without limitation, bone pain, anemia, acute renal failure, high serum proteins, and multiple electrolyte disturbances. Also, in multiple myeloma, hypercalcemia is observed in approximately one-third of patients and could be mediated by parathyroid hormone-related peptide, osteolytic cytokine production, and excess 1,25-dihydroxyvitamin D production [3, 4]. However, in asymptomatic patients, it is important that pseudohypercalcemia associated to paraproteins is excluded; thus, ionized calcium measurement is recommended. On the contrary, hyperphosphatemia is most likely secondary to renal dysfunction. Nonetheless, hypophosphatemia is rare, and there are few reported cases. For instance, Mao and Ong described a case of hypophosphatemia that was due to paraproteins causing assay interference [5]. As you will see in our case, we faced the classic presentation of multiple myeloma with subsequent development of dangerous hypophosphatemia during medical regimen.

## 2. Case Report

This is the case of a 56-year-old Hispanic woman with a past medical history of arterial hypertension, type 2 diabetes mellitus, and chronic anemia who was transferred from a community medical clinic to our institution for management of symptomatic anemia, severe hypercalcemia, and renal failure. Upon evaluation at our institution, the patient stated that, for the last three months, she had been experiencing fatigue, generalized weakness, back pain, decreased appetite, and unquantified weight loss. Moreover, the day prior to admission, she had an episode of lightheadedness followed by loss of consciousness for which she was taken to the community medical clinic for evaluation. Upon arrival to our institution, her blood pressure was 161/76 mmHg with a heart rate of 78 bpm, respiratory rate of 18 rpm, temperature of 36.5°C, and peripheral oxygen saturation of 99% at room air. The physical examination provided a remarkable perspective for an acutely ill appearance, generalized paleness, and dry oral mucosa. Initial laboratories were also remarkable for normocytic normochromic anemia of 6.5 g/dL (12.0–14.0), albumin-corrected calcium of 13.8 mg/dL (8.8–10.3), and elevated creatinine of 6.7 mg/dL (0.60–1.10) with a glomerular filtration rate of 7 min/mL (>60). 25-Hydroxyvitamin D was within normal limits at 38.1 ng/ml (30.0–100.0), whereas 1,25-dihydroxyvitamin D was low at 8.6 pg/mL (19.9–79.3). Notwithstanding, the patient had normal phosphorus level in 2.50 mg/dL (2.40–4.20) and normal intact parathyroid hormone in 25 pg/mL (11–67) (see Table 1). Finally, radiographic images disclosed multiple skeletal lytic lesions. In light of such clinical presentation, the patient was admitted into the internal medicine ward with ongoing diagnosis of symptomatic anemia, acute renal failure, and moderate hypercalcemia.

During the early course of hospitalization, both nephrology and hematology/oncology services were consulted for further recommendations regarding renal function deterioration with multiple electrolyte disturbances and a high suspicion of multiple myeloma. The patient was initially managed with aggressive intravenous hydration with isotonic saline and systemic dexamethasone, with marked improvement in renal function during the course of the first week. The results from bone marrow biopsy were consistent with high-risk IgG/kappa multiple myeloma, reason why she was started on chemotherapy with cyclophosphamide, bortezomib, and dexamethasone (CyBorD).

At this point, it was remarkable that the patient continued with corrected hypercalcemia of 11 mg/dL (8.8–10.3) and developed hypophosphatemia of 1.7 mg/dL (2.40–4.20). During the second week of hospitalization, the patient suffered a pathological fracture of the right humerus for which a single dose of zoledronic acid was administered. Therefore, based on the evidence of the pathological fracture, the patient was evaluated by radiation-oncology specialists and was treated with external beam radiotherapy (IMRT technique) up to about the total dose of 4000 cGy.

Furthermore, at day 20, endocrinology service was consulted for recommendations regarding persistent hypophosphatemia despite oral and intravenous replacement. Consequently, new laboratory workup was ordered and was remarkable for marked elevation of intact PTH in 711 pg/ml (11–67), 1,25-dihydroxyvitamin D of 123.0 pg/mL (19.9–79.3), normal albumin-corrected calcium of 8.9 mg/dl (8.8–10.3), and fractional excretion of phosphorus in 14% with reference range <5%. The ionized calcium level was low at 1.15 mEq/L (1.17–1.58) (see Table 2) and phosphorus in 1.4 mg/dl (2.40–4.20).

In view of the persistent hypophosphatemia and the new laboratory results, we considered secondary hyperparathyroidism and pseudohypercalcemia as the etiology of hypophosphatemia. The patient was initiated on calcitriol, and as a result, electrolytes normalized. She was discharged from our institution after 30 days of hospitalization, with remarkable laboratories at the moment of discharge for normal renal function with creatinine at 0.96 mg/dL (0.6–1.10), albumin-corrected calcium at 9.9 mg/dL (8.8–10.3), and phosphorus at 4.70 mg/dL (2.40–4.20) (see Table 3). Eventually, the patient was successfully treated with an autologous stem cell transplant with excellent response.

## 3. Discussion

Patients with malignancies are exposed to experience multiple metabolic abnormalities. These disturbances can be part of the original process of the disease, medical treatment, or complications during the progression of the disease.

Multiple myeloma (MM) is characterized by proliferation of monoclonal plasma cells in the bone marrow, resulting thereby in overproduction of monoclonal paraproteins. The excessive plasma cell production results in extensive skeletal destruction with osteolytic lesions and pathologic fractures. Also, the deposition of paraproteins in kidneys affecting the renal function coupled with the lytic lesions can provoke the most common electrolyte disturbances seen in MM, which are hypercalcemia and hyperphosphatemia.

In our case, the patient was diagnosed with multiple myeloma with a typical presentation of the disease. During the first weeks of hospitalization, albumin-corrected calcium showed elevated values until ionized calcium ordered during the workup for persistent hypophosphatemia demonstrated the opposite. This finding coupled with the fact that the patient did not present symptoms of hypercalcemia favored the diagnosis of pseudohypercalcemia.

There is evidence that multiple myeloma can occasionally cause laboratory artifacts due to paraproteinemia, causing thereby confusion among physicians. For instance, Ashrafi et al. and Caras reported laboratory interference in electrolytes due to high levels of paraproteins [6, 7]. The proposed mechanisms of these findings include binding of calcium to abnormal immunoglobulins interfering with immunologic assays [4].

Initially, the patient presented normal intact parathyroid hormone (PTH), which exponentially increased after management of factitious hypercalcemia in multiple myeloma. In the absence of high ionized calcium, elevated PTH is the equivalent to secondary hyperparathyroidism [8]. The main mechanism that explains the secondary hyperparathyroidism in our patient is the expected response to true hypocalcemia. As part of the dynamic of parathyroid secretion in response to the ongoing hypocalcemia and improvement in the renal function, intact PTH and 1,25-dihydroxyvitamin D increased significantly provoking the dramatic changes in the homeostasis of phosphorus, which was observed in our case.

In addition, the patient developed severe hypophosphatemia with persistent inadequate response to medical replacement. The effects of elevated parathyroid hormone (PTH) decreased the activity of sodium phosphate cotransporters at the proximal and distal renal tubules, causing an increase in phosphate excretion [9, 10]. This mechanism was evident in our patient with elevated fractional excretion of phosphate during the course of medical management. The primary cause of transient hypophosphatemia in our patient was the secondary hyperparathyroidism caused by true low ionized calcium.

Moreover, several medical therapies utilized in multiple myeloma including, without limitation, volume expansion, bisphosphonates, and corticosteroids, are mainly associated to the increase in phosphorus excretion. Consequently, the patient was treated with a single dose of zoledronic acid infusion, which is a bisphosphonic acid that inhibits the resorption and remodeling activity of the bones. The manufacturer prescribing information of zoledronic acid describes the incidence of hypophosphatemia in 973 patients treated for multiple myeloma and bone metastasis of solid tumors. Grade 3 hypophosphatemia (serum phosphate less than 2 mg/dL) was 12%, while grade 4 hypophosphatemia (serum phosphate less than 1 mg/dL) was less than 1%. Thus, although the exact mechanism of zoledronic acid-induced hypophosphatemia is not completely understood, the hypothesized explanations are that the adverse effect of hypocalcemia provokes induced hyperparathyroidism aggravating the phosphate excretion. This could be a possibility in our case. However, we need to point out the fact that the 973 patients had a median duration of exposure for a safety period of 12.8 months with zoledronic acid every 3-4 weeks, whereas in our case, the patient was treated with a single dose of zoledronic acid. As mentioned, the combined treatments for factitious hypercalcemia coupled with the original process and complications of the disease can trigger electrolyte disorders that could be very challenging to treat.

## 4. Conclusion

In summary, this case report highlights different uncommon electrolyte disorders that can be found in patients with multiple myeloma. Pseudohypercalcemia in the setting of elevated total proteins should always be taken under consideration, particularly when the patient does not exhibit signs and symptoms suggestive of elevated calcium level. In such cases, measurement of ionized calcium should be strongly considered to determine the true extracellular calcium levels. Additionally, the combined treatment for factitious hypercalcemia and renal impairment can trigger secondary hyperparathyroidism aggravating thereby other electrolyte disorders.

Finally, the multiple electrolyte disturbances faced in multiple myeloma and medication side effects can precipitate serious metabolic disorders such as hypophosphatemia seen in our patient. There are different mechanisms including an increase in phosphate excretion and intracellular shifting worsening the phosphate level. For that reason, the physician should be able to differentiate between true and spurious electrolyte imbalance in order to prevent unnecessary interventions that could be detrimental to the patient.

## Figures and Tables

**Table 1:** Laboratories upon admission.

BUN (mg/dL)	Creatinine (mg/dL)	Calcium (mg/dL)	Albumin (g/dL)	Total protein (g/dL)	Magnesium (mg/dL)	Phosphorous (mg/dL)	Intact PTH (pg/ml)	1,25-Dihydroxyvitamin D (pg/ml)	25-Hydroxyvitamin D (ng/ml)
67.00	7.00	12.40	2.30	11.50	2.06	2.50	25	8.6	38.1
(8–21)	(0.6–1.10)	(8.8–10.3)	(3.7–4.9)	(6.2–7.9)	(1.8–2.2)	(2.4–4.2)	(11–67)	(19.9–79.3)	(30–100)

**Table 2:** Laboratories at the moment of endocrinology service evaluation.

BUN (mg/dL)	Creatinine (mg/dL)	Calcium (mg/dL)	Albumin (g/dL)	Total protein (g/dL)	Magnesium (mg/dL)	Phosphorous (mg/dL)	Intact PTH (pg/ml)	1,25-Dihroxyvitamin D (pg/ml)	1,25-Dihydroxyvitamin D (ng/ml)	Ionized calcium (mEq/L)
17.0	1.13	7.50	2.20	8.60	1.73	1.40	711	123	31.7	1.15
(8–21)	(0.6–1.10)	(8.8–10.3)	(3.7–4.9)	(6.2–7.9)	(1.8–2.2)	(2.4–4.2)	(11–67)	(19.9–79.3)	(30–100)	(1.17–1.58)

**Table 3:** Laboratories at the moment of discharge.

BUN (mg/dL)	Creatinine (mg/dL)	Calcium (mg/dL)	Albumin (g/dL)	Total protein (g/dL)	Magnesium (mg/dL)	Phosphorous (mg/dL)
8.00	0.96	8.50	2.30	7.80	1.52	4.70
(8–21)	(0.6–1.10)	(8.8–10.3)	(3.7–4.9)	(6.2–7.9)	(1.8–2.2)	(2.4–4.2)

## Data Availability

No data were used to support this study.
